# Engineering of a microbial coculture of *Escherichia coli* strains for the biosynthesis of resveratrol

**DOI:** 10.1186/s12934-016-0562-z

**Published:** 2016-09-29

**Authors:** José M. Camacho-Zaragoza, Georgina Hernández-Chávez, Fabian Moreno-Avitia, René Ramírez-Iñiguez, Alfredo Martínez, Francisco Bolívar, Guillermo Gosset

**Affiliations:** Departamento de Ingeniería Celular y Biocatálisis, Instituto de Biotecnología, Universidad Nacional Autónoma de México, Apdo. Postal 510-3, 62210 Cuernavaca, Morelos Mexico

**Keywords:** Metabolic engineering, *Escherichia coli*, Microbial coculture, Resveratrol, Glycerol

## Abstract

**Background:**

Resveratrol is a plant natural product with many health-protecting effects which makes it an attractive chemical both for academic studies and industrial purposes. However, the low quantities naturally produced by plants as well as the unsustainable procedures of extraction, purification and concentration have prompted many biotechnological approaches to produce this chemical in large quantities from renewable sources. None of these approaches have considered a microbial coculture strategy to produce this compound. The aim of this study was to prove the functionality of a microbial coculture for the biosynthesis of resveratrol.

**Results:**

In this work, we have successfully applied a coculture system strategy comprised of two populations of *Escherichia coli* strains, each with a partial and complementary section of the pathway leading to the biosynthesis of the stilbene resveratrol. The first strain is a *pheA* knockout mutant previously engineered to excrete p-coumaric acid into the medium through the overexpression of genes encoding a tyrosine ammonia lyase from *Rhodothorula glutinis*, a feedback resistant 3-deoxy-D-*arabino*-heptulosonate 7-phosphate synthase and a transketolase. The second strain in the coculture was engineered to express the second part of the resveratrol biosynthetic pathway through the introduction of synthetic genes encoding the 4-coumaroyl-CoA ligase from *Streptomyces coelicolor* A2 and the stilbene synthase either from the peanut *Arachis hypogaea* or the grapevine *Vitis vinifera,* the latter synthesized employing a gene harmonization strategy and showing better resveratrol production performance. Batch cultures were performed in mineral medium with glycerol as the sole carbon source, where a final titer of 22.6 mg/L of resveratrol was produced in 30 h.

**Conclusions:**

To our knowledge, this is the first time that a coculture of bacterial strains is used for the biosynthesis of resveratrol from glycerol, having the potential for a greater improvement in the product yield and avoiding the use of precursors such as p-coumaric acid, yeast extract or an expensive inhibitor such as cerulenin.

## Background

Stilbenes are natural products from the phenylpropanoid family displaying extensive diversity of chemical structures and biological activities. The stilbene resveratrol can be found in red wine, and together with other phenolic compounds, it has been suggested to reduce the risk of coronary heart disease [1]. Even though more studies are needed to verify the cardioprotective effects of resveratrol in vivo, some studies have shown that resveratrol has potent antioxidant properties and inhibits lipid peroxidation, hence, protecting LDL molecules and contributing to prevent atherosclerosis [2]. Antitumor, neuroprotective, hypoglycemic and antimicrobial are some other effects that have also been attributed to resveratrol [3–7].

**Fig. 1:**
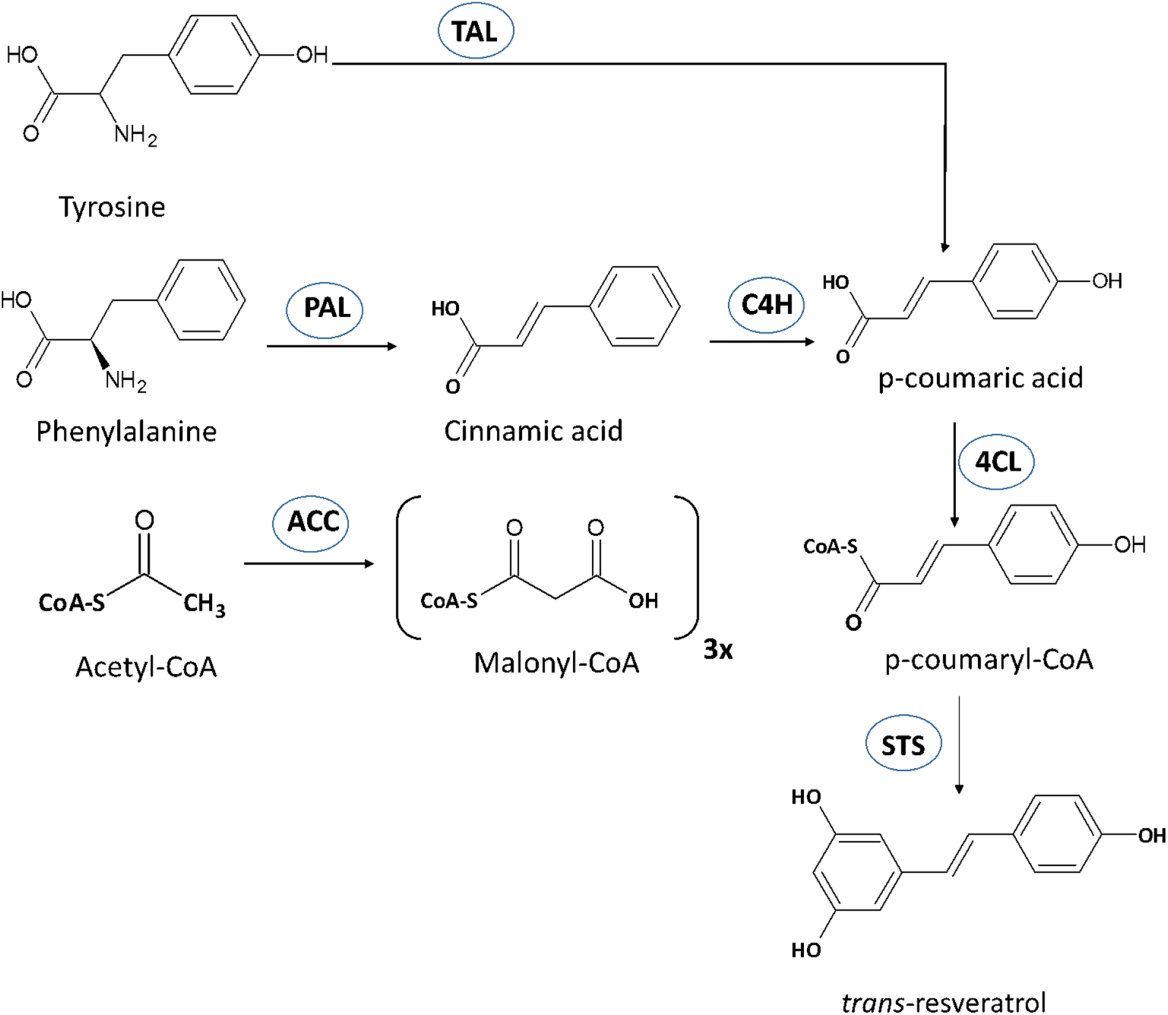
Map of the trans-resveratrol biosynthetic pathway. *TAL*, tyrosine ammonia lyase, *PAL* phenylalanine ammonia lyase, *C4H* cinnamate 4-hydroxylase, *4CL* 4-coumaroyl-CoA ligase, *STS* stilbene synthase, *ACC* acetyl-CoA carboxylase

The first step in the biosynthesis of resveratrol is the transformation of l-phenylalanine or l-tyrosine into p-coumaric acid, which can proceed in two possible routes (Fig. 1); The first one is through the reactions catalyzed by l-phenylalanine ammonia lyase (PAL) followed by the action of the cinnamate 4-hydroxylase (C4H); and the second one is through the sole action of the enzyme l-tyrosine ammonia lyase (TAL). C4H is a cytochrome P450 enzyme that requires the co-expression of a membrane-bound cytochrome P450 reductase for its full functionality. The second step in resveratrol synthesis consists in the activation of p-coumaric acid through its esterification with one molecule of coenzyme A (CoA), which is performed by the enzyme 4-coumaroyl-CoA ligase (4CL), yielding p-coumaroyl-CoA. This is the starter molecule required by the last enzyme, stilbene synthase (STS), which catalyzes the condensation of one molecule of p-coumaroyl-CoA and three units of malonyl-CoA through a series iterative decarboxylating reactions that finish with the cyclization of a tetraketide intermediate, resulting in one molecule of trans-resverartrol. So far, many STS genes have been described from plants such as *Vitis vinifera, Arachis hypogea, Fallopia japonica (syn. Polygonum cuspidatum), Rheum tataricum, Parthenocissus henryana, Pinus, Sorghum*, *Petroselinum crispum* and more, extending the repertoire of enzymes that can be used for metabolic engineering of heterologous hosts.

Several strategies have been applied for the microbial production of resveratrol in *Escherichia coli*. An essential modification involves the heterologous expression of the genes coding for 4CL and STS enzymes. Strains expressing these genes have the capacity of converting supplemented p-coumaric acid into resveratrol [8]. Also, if a gene encoding for a TAL activity is expressed, then the strain is able to use supplemented l-tyrosine as a precursor for resveratrol [9]. The amino acid l-phenylalanine could also be employed as a precursor, however, since the required expression of the P450 reductase is deficient in *E. coli*; this approach has not been successful [10]. Other additional modifications have been used to increase the availability of the precursor malonyl-CoA, such as the introduction of the genes *matB* and *matC* from *Rhizobium leguminosarum bv. trifolii* which encode a malonyl-CoA synthetase and a malonate transporter protein, respectively. These activities can increase the concentration of malonyl-CoA when cells are cultured in medium supplemented with malonate [9, 11, 12]. An alternative approach to increase malonyl-CoA involves the utilization of cerulenin. This compound is an inhibitor of the *fabB* and *fabF* gene products which consume malonyl-CoA for lipids biosynthesis. Although cerulenin addition has a clear positive effect on resveratrol production [13], the high cost of the inhibitor makes this strategy impractical. These and other strategies have been applied for optimization of *E. coli* strains and for improving the culture conditions, yielding resveratrol titers from 3.6 to 2340 mg/L (Table 1).

**Table 1:** Comparison of reports on resveratrol production using *E. coli* strains

Microorganism	Precursor	Time of culture/media	Genes introduced	Genes origin	Production (mg/L)	Reference
*E. coli*	p-coumaric acid	24 h, 2 × YT	*4CL, STS*	*N. tabacum (4CL), V. vinifera (STS)*	16	[29]
*E. coli*	p-coumaric acid	19 h, M9 + yeast extract + glycerol	*4CL, STS*	*A. thaliana (4CL), A. hypogaea (STS)*	104.5	[8]
*E. coli*	p-coumaric acid	72 h, minimal MOPS medium + glucose	*4CL, STS*	*S. coelicolor (4CL), V. vinifera (STS)*	3.6	[30]
*E. coli*	p-coumaric acid	60 h, M9 + yeast extract + glucose	*4CL, STS*	*Lithospermum erythrohizon (4CL), A. hypogaea (STS)*	171	[31]
*E. coli*	p-coumaric acid	24 h, M9 + yeast extract + glycerol, cerulenin	*4CL, STS*	*A. thaliana (4CL), V. vinifera (STS)*	2340	[13]
*E. coli*	l-tyrosine, malonate	48 h, minimal MOPS medium + glucose	*TAL, 4CL, STS, MatB, MatC*	*R. glutinis (TAL), Petroselinum crispum (4CL), V. vinifera (STS), R. trifoli (matB, matC)*	35	[9]
*E. coli*	p-coumaric acid	20 h, M9 + glycerol	*4CL, STS*	*S. coelicolor (4CL), V. vinifera (STS)*	78.1	This work
*E. coli/E.coli pheA*- *(Coculture)*		20 h, M9 + glycerol + l-Phe	*TAL, 4CL, STS*	*R. glutinis (TAL), S. coelicolor (4CL), V. vinifera (STS)*	22	This work

In contrast to the rather artificial conditions used in industrial production processes, microbial species usually exist in Nature as part of a consortium. The members of a consortium contribute with specialized metabolic functions that help in surviving adverse and changing conditions. In biotechnological processes, consortia have been employed traditionally in bioremediation and water treatment applications. However, until recently, the simultaneous culture of two distinct metabolically engineered strains (coculture) was not used for the production of useful compounds. This approach can reduce the metabolic burden of each strain and allow for parallel optimization of the engineered pathway in a modular fashion. Distribution of a biochemical pathway into two strains also has the advantage of compartmentalization, preventing potential inhibiting metabolic intermediates from acting upon susceptible enzymes [14, 15]. Successful coculture strategies have been reported for cis,cis-muconic acid production in engineered *E. coli* strains [16, 17]. In these reports an improvement in production capacity of the coculture when compared to monoculture processes has been demonstrated [16, 17]. In another example, a coculture approach was employed to optimize flavan-3-ols production from phenylpropanoic acid precursors [18]. The six-step biosynthetic pathway leading to (+)-afzelechin and (+)-catechin was divided among two *E. coli* strains and strain optimization was complemented with computer-assisted process optimization considering variables such as carbon source, gene induction, temperature and inoculation ratio. The final optimized system resulted in a 970-fold improvement in product titer over previous reports of monocultures [18].

**Fig. 2:**
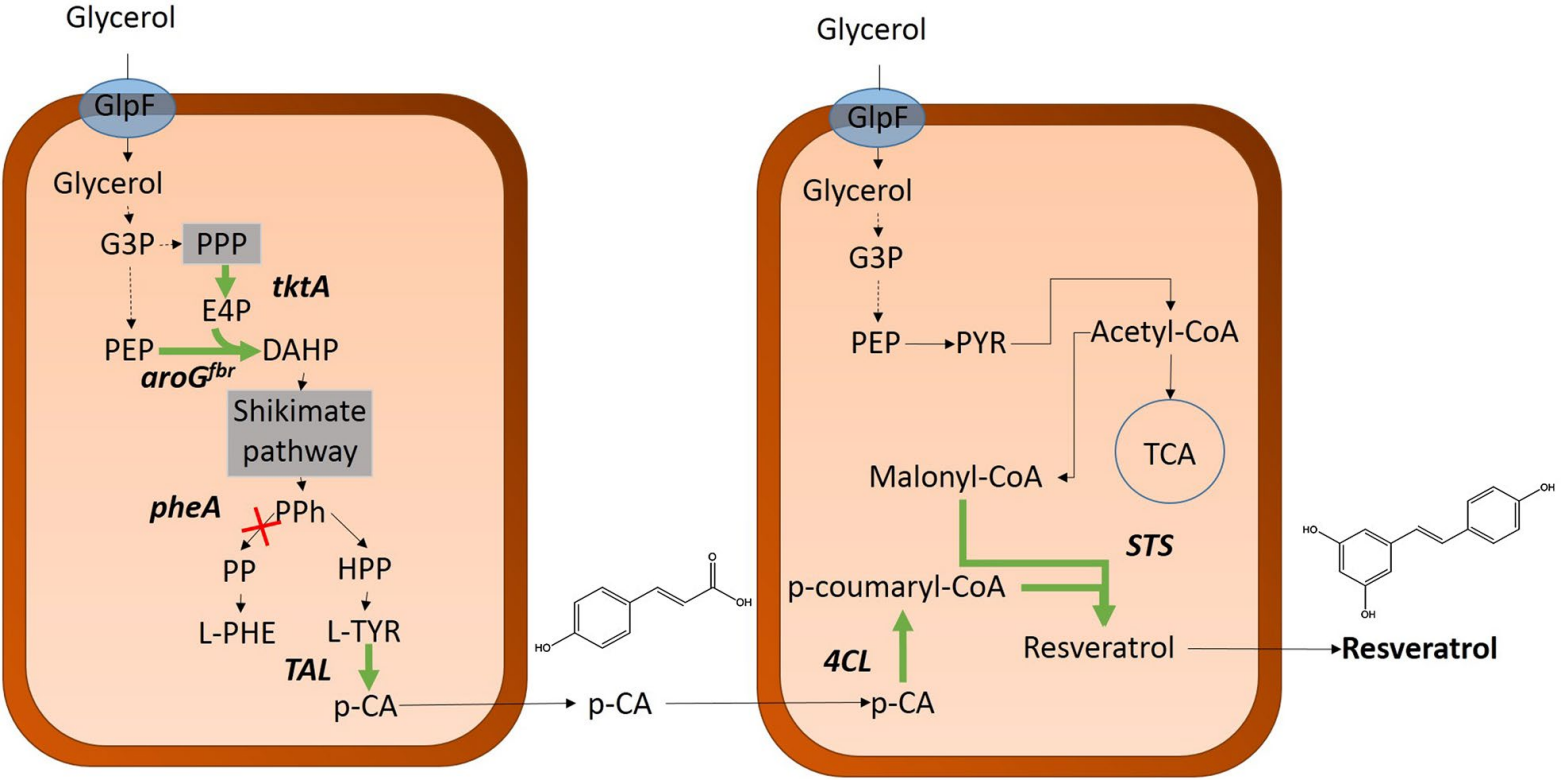
Schematic diagram of the coculture system proposed for the biosynthesis of resveratrol. Episomal expression of the correspondent genes is represented by *thicker green arrows* and *bold* names, whereas the *red cross* indicates inactivation of the corresponding gene in *bold*. *G3P* glyceraldehyde-3-phosphate, *PEP* phosphoenolpyruvate, *E4P* erithrose 4-phosphate, *DHAP* 3-deoxy-d-*arabino*-heptulosonate 7-phosphate, *PPh* prephenate, *PP* phenylpyruvate, *HPP* hydroxyphenylpyruvate, *L-PHE* L-phenylalanine, *L-TYR* L-tyrosine, *p-CA* p-coumaric acid, *PYR* pyruvate

For the case of resveratrol biosynthesis, the strategies to optimize the carbon flux towards the aromatic amino acids biosynthetic pathway could hinder optimization strategies to simultaneously increase the pool of malonyl-CoA because of the divergent nature of these pathways. To overcome this issue, a coculture approach might represent a better choice, allowing for individual design and optimization of two strains; each one specialized to efficiently provide one of the precursors of the heterologous pathway, following the principle of an assembly line based on stages. Accordingly, the resveratrol biosynthetic pathway can be divided into two parts; on one hand, harnessing the metabolic machinery of one strain to synthesize p-coumaric acid and excrete it to the medium, and then a second engineered strain able to efficiently provide the acyl extender units of malonyl-CoA to the STS and finally yielding resveratrol. Furthermore, it has been suggested that TAL enzymes could be inhibited by coumaroyl-CoA [12]. Therefore, by splitting the pathway into two separate strains, this potential inhibition could be prevented, given the artificial compartmentalization of coumaroyl-CoA and TAL (Fig. 2). In this work, we show the feasibility of dividing the resveratrol biosynthetic pathway among two different *E. coli* strains to produce this compound in a coculture.

## Results and discussion

### Selection of 4CL and STS enzymes and characterization of resveratrol biosynthesis in minimal medium with supplementation of p-coumaric acid

As an initial step to generate a bacterial platform for the biosynthesis of resveratrol, we first built a plasmid for expressing the genes coding for the last two enzymes required by the biosynthetic pathway: 4CL and STS. We chose to express the 4CL from *Streptomyces coelicolor* A2 given its microbial origin and its high catalytic efficiency compared to other homologous enzymes [19]. In the case of STS, we decided to test the performance of enzymes from two distinct origins: *A. hypogaea* (AhSTS) and *V. vinifera* (VvSTS), since previous studies have shown that these proteins display better catalytic properties compared to others [13]. The transformed resultant *E. coli* W3110 strains were named W-Ah and W-Vv, respectively. These strains were cultured in M9 minimal medium supplemented with glucose or glycerol, and we added p-coumaric acid as a precursor for resveratrol, given that these strains do not express a TAL enzyme. In this experiment, we tested the expression and functionality of the plasmid-encoded proteins in an environment non-limited for one of the two resveratrol precursors (p-coumaric acid).

**Fig. 3:**
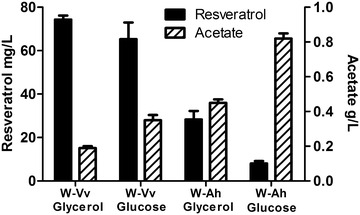
Resveratrol titers and acetate production. Final titers of resveratrol and acetate after 30 h of culture in M9 mineral medium supplemented with glycerol or glucose and p-coumaric acid. Vv, STS from *V. vinifera;* Ah, STS from *A. hypogaea*. *Error bars* represent standard deviation

**Fig. 4:**
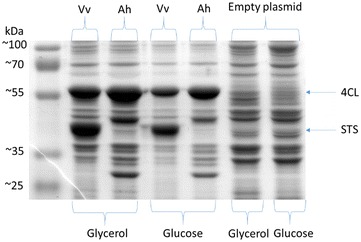
Effect of the carbon source and enzyme origin on the protein levels of 4CL and STS. 12 % SDS-PAGE stained with Coomasie blue. 4CL = 55.3 kDa; AhSTS = 42.8 kDa; VvSTS = 42.8 kDa

We set up shake flask cultures and induced the expression of the plasmid-encoded enzymes by adding 0.5 mM of IPTG when cells reached an O.D._600_ of 0.8. The growth kinetic parameters are shown in Table 2. Our results showed that the use of glycerol as a carbon source causes a higher resveratrol production when compared to glucose (Fig. 3; Table 3). When we compared the final resveratrol titers as a function of the carbon source, strain W-Vv showed an almost 16 % increase, while the strain W-Ah showed an even larger increase of about threefold when grown in glycerol compared to glucose (Table 3). The higher difference observed in the case of W-Ah compared to W-Vv could be a consequence of the distinct catalytic properties of both enzymes (AhSTS and VvSTS) and their expression levels. Enzyme VvSTS is highly expressed and has around twice the turnover number when compared to the enzyme from *A. hypogaea* (Fig. 4) [13]. These two features could explain the observed results.

**Table 2:** Growth kinetic parameters for monocultures in M9 mineral medium, 30 °C

Strain	Substrate	µ (h^−1^)	X_MAX_ (g/L)	Y_X/S_ (g_DCW_/g_S_)	q_S_^a^ (g_S_/g_DCW_ h)
W/AhSTS	GLC	0.25 ± 0.01	1.32 ± 0.02	0.32 ± 0.08	0.79 ± 0.20
W/AhSTS	GLY	0.18 ± 0.01	1.11 ± 0.00	0.45 ± 0.25	0.48 ± 0.19
W/VvSTS	GLC	0.29 ± 0.01	1.48 ± 0.03	0.48 ± 0.07	0.60 ± 0.06
W/VvSTS	GLY	0.18 ± 0.00	1.16 ± 0.04	0.17 ± 0.02	1.12 ± 0.10

*GLC* glucose, *GLY* glycerol

^a^Calculated from the exponential growth phase

**Table 3:** Resveratrol production parameters for monocultures in M9 mineral medium, 30 °C

Strain	Substrate	Y_R/S_ (mg_P_/g_S_)	Y_P/X_ (mg_p_/g_DCW_)	Q_P_ (mg_p_/L h)	Resveratrol (mg/L)
W/AhSTS	GLC	1.83 ± 0.26	7.78 ± 1.02	0.40 ± 0.06	8.04 ± 1.11
W/AhSTS	GLY	11.75 ± 1.53	34.86 ± 5.48	1.42 ± 0.20	28.31 ± 3.93
W/VvSTS	GLC	13.52 ± 0.34	55.16 ± 4.77	3.26 ± 0.39	65.28 ± 7.70
W/VvSTS	GLY	39.44 ± 1.77	84.51 ± 2.92	3.71 ± 0.09	74.30 ± 1.84

*GLC* glucose, *GLY* glycerol

Clear differences in resveratrol production were also observed concerning the origin of the STS enzyme encoded in each plasmid vector. In shake-flask cultures grown in glycerol, strain W-Vv reached 2.8-fold higher resveratrol titers than strain W-Ah (Table 3). In glucose supplemented medium, the difference was even more evident, since resveratrol titers in strain W-Vv were about sevenfold higher than the strain W-Ah (Table 3).

We performed an SDS-PAGE stained with Coomassie blue to determine the relative protein levels of the enzymes 4CL and STS at the end of the production cultures (Fig. 4). Lower levels of all the heterologous enzymes were observed in cultures grown on glucose compared to glycerol. The levels of 4CL displayed a slightly lower expression in glucose compared to glycerol. These results suggest that a physiological component of the expression apparatus might be sensitive to the nature of the carbon source, affecting the synthesis of these proteins. However, the most evident difference regarding the protein levels of the heterologous enzymes was related to the origin of the expressed genes. We found that the levels of VsSTS were significantly higher than the levels of AhSTS (Fig. 4). Further studies are needed to establish whether the differences in the expression of these two enzymes is a consequence of dissimilarities in the stability of the RNA transcript, the translation efficiency or the protein stability. These results agree well with the production levels of resveratrol as described above (Table 3) and suggests that the higher expression of the heterologous proteins in cultures grown in glycerol and the higher expression of the STS from *V. vinifera* accounts for the higher resveratrol titers observed when using this carbon source. A possible explanation for this observation is that a higher enzyme activity due to the higher abundance of the stilbene synthase could help in driving the carbon flux from malonyl-CoA towards the synthesis of resveratrol. An alternative explanation arises from the analysis of acetate metabolism and its relation with acetyl-CoA. The inhibition of STS by acetyl-CoA has been studied by the group of Koffas et al. [13]. They reported an apparent competitive inhibition constant of about 460 μM, triggered by acetyl-CoA against STS activity from *V. vinifera* [13]. However, they assumed that the intracellular levels of acetyl-CoA in *E. coli* cells were not as high to produce a significant inhibition of STS. Nevertheless, according to Takamura et al., under aerobic and glucose growth conditions, the acetyl-CoA concentration in *E. coli* cells range from 20–600 μM [20]. Therefore, it is possible that the intracellular concentration of acetyl-CoA exerts a substantial inhibitory effect on the STS. In support for this hypothesis, we found an inverse relation between the titers of resveratrol and the amount of acetate accumulated in the culture medium (Fig. 3, Tables 3 and 4). The higher content of acetate in glucose-grown cultures agrees with the previous observation that glucose-grown cells have higher intracellular acetyl-CoA levels than cells grown in glycerol, given that acetyl-CoA is a direct precursor of acetate [20]. However, other factors such as a different carbon flux distribution, the loss of carbon in acetate formation and the potential toxic effect of this organic acid, could also contribute to the observed differences in resveratrol production when comparing cultures in glucose with respect to glycerol.

**Table 4:** Acetate production parameters for monocultures in M9 mineral medium, 30 °C

Strain	Substrate	Y_P/S_ (g_P_/g_S_)	Y_P/X_ (g_p_/g_DCW_)	q_P_^a^ (g_P_/g_DCW_ h)	Acetate (g/L)
W/AhSTS	GLC	0.13 ± 0.03	0.41 ± 0.02	0.10 ± 0.01	0.82 ± 0.03
W/AhSTS	GLY	0.04 ± 0.02	0.10 ± 0.01	0.02 ± 0.00	0.45 ± 0.02
W/VvSTS	GLC	0.15 ± 0.03	0.30 ± 0.02	0.09 ± 0.01	0.35 ± 0.03
W/VvSTS	GLY	0.01 ± 0.01	0.05 ± 0.06	0.01 ± 0.01	0.19 ± 0.01

*GLC* glucose, *GLY* glycerol

^a^Calculated from the exponential growth phase

Additionally, as was suggested by Watts et al. [8] the transport of phenylpropanoids into the cytoplasm of *E. coli* could be inhibited in cells growing in glucose due to the catabolic repression of the operons *hca and mhp* involved in the transport and catabolism of these compounds. Therefore, cell cultures supplemented with glycerol instead of glucose could help in the maintenance of a constant flux of p-coumaric acid into the cells, driving forward resveratrol biosynthesis.

### Characterization of monocultures for the production of p-coumaric acid and resveratrol

The following experiments were performed with a strain expressing the STS from *V. vinifera* since higher resveratrol titers were produced by the *E. coli* strain expressing the gene encoding this enzyme. Previously in our group, we reported an engineered strain for the production of p-coumaric acid [21]. This strain harbors a plasmid encoding the TAL enzyme from the yeast *Rhodothorula glutinis* and another plasmid with the *E. coli* genes *aroG*^*fbr*^ and *tktA* encoding a feedback-inhibition-resistant 3-deoxy-D-*arabino*-heptulosonate 7-phosphate (DAHP) synthase and a transketolase, respectively. Furthermore, to improve l-tyrosine accumulation, in this strain the *pheA* gene which encodes for a chorismate mutase/prephenate dehydratase, was inactivated. The enzyme TAL from *R. glutinis* was selected since it showed a higher affinity towards l-tyrosine rather than l-phenylalanine [21]. This *E. coli* strain was named W(pheA-)Rg.

**Fig. 5:**
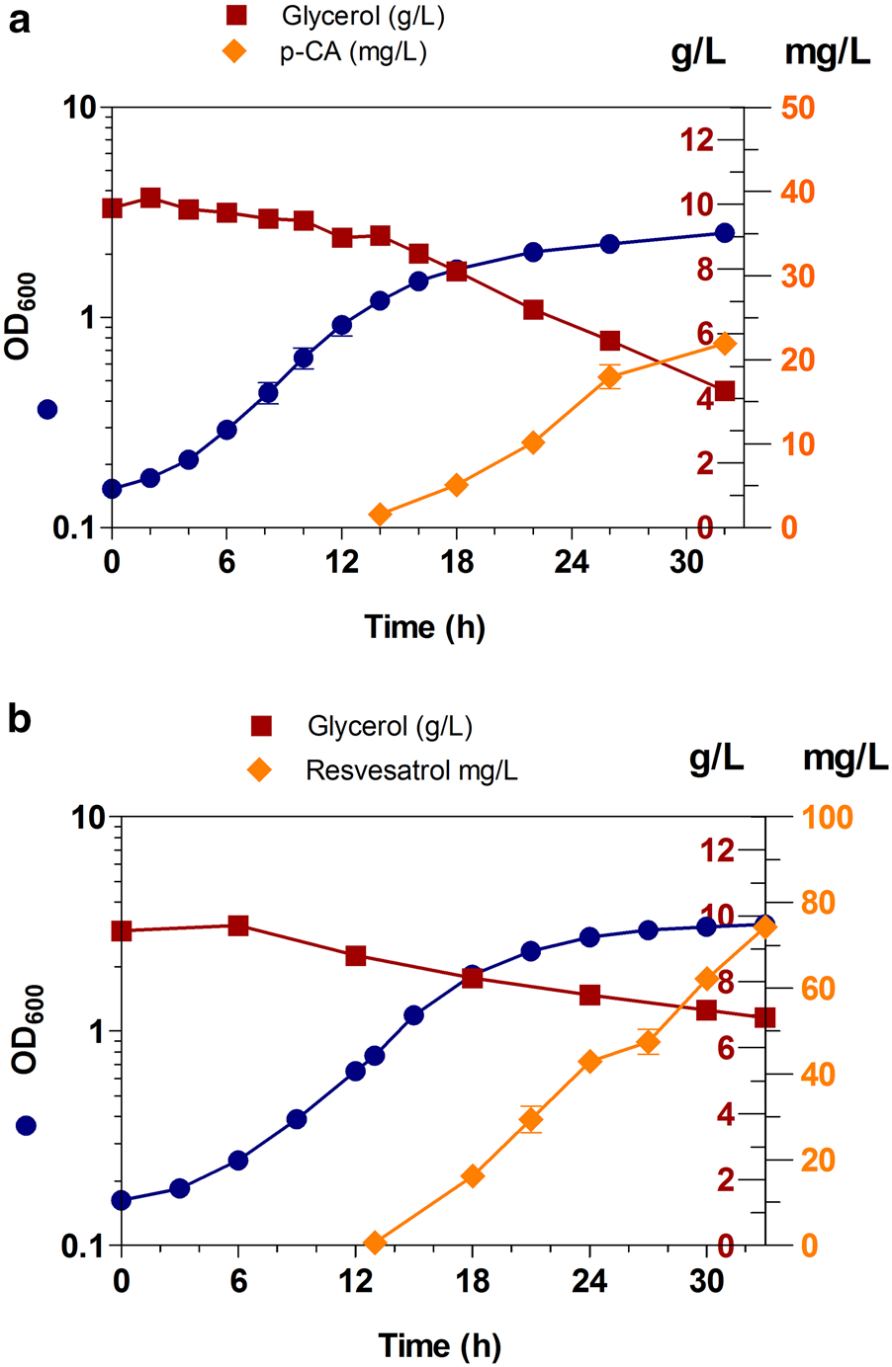
Growth kinetics and production profiles of monocultures. Production of p-coumaric acid (**a**) or resveratrol (**b**) in M9 mineral medium supplemented with glycerol. *Error bars* represent standard deviation

To characterize strains W(pheA-)Rg and W-Vv, we performed shake-flask cultures individually with each strain in M9 mineral salts medium supplemented with glycerol as the sole carbon source. The results showed that both strains display similar specific growth rates (Fig. 5a, b) (Table 5). When compared to W(pheA-)Rg, strain W-Vv reached around a 20 % higher biomass, but the consumption rate of glycerol appeared lower. The production of p-coumaric acid by strain [W(pheA^−^)Rg] reached 133 uM (~22 mg/L) and resveratrol from W-Vv, upon addition of 3 mM of p-coumaric acid, reached ~78.1 mg/L (Fig. 5b). These results are the highest resveratrol titers reported with an *E. coli* recombinant strain cultured in mineral medium without yeast extract supplementation.

**Table 5:** Growth and p-coumaric acid production parameters of W(pheA^−^)Rg and W-Vv in glycerol M9 mineral medium, 30 °C

Strain	µ (h^−1^)	X_MAX_ (g/L)	Y_X/S_ (g_DCW_/g_S_)	q_S_^a^ (g_S_/g_DCW_ h)
W(pheA^−^)Rg	0.19 ± 0.00	0.93 ± 0.04	0.62 ± 0.34	0.37 ± 0.18
W/VvSTS	0.18 ± 0.01	1.16 ± 0.04	0.17 ± 0.02	1.12 ± 0.10

	Y_P/S_ (mg_P_/g_S_)	Y_P/X_ (mg_p_/g_DCW_)	Q_P_ (mg_p_/L h)	p-CA (mg/L)
W(pheA^−^)Rg	4.63 ± 0.25	34.12 ± 2.91	1.10 ± 0.03	21.91 ± 0.64

^a^Calculated from the exponential growth phase

### Coculture in M9 mineral medium for the production of resveratrol from glycerol

To determine the feasibility of reconstituting a fully functional biosynthetic pathway to produce resveratrol, the strain with the capacity of transforming glycerol to p-coumaric acid (W(pheA-)Rg) and the strain able to convert it to resveratrol (W-Vv), were both grown in a coculture in M9 mineral medium supplemented with glycerol as the sole carbon source. It is important to point out that neither p-coumaric acid nor yeast extract were supplemented to the culture medium.

**Fig. 6:**
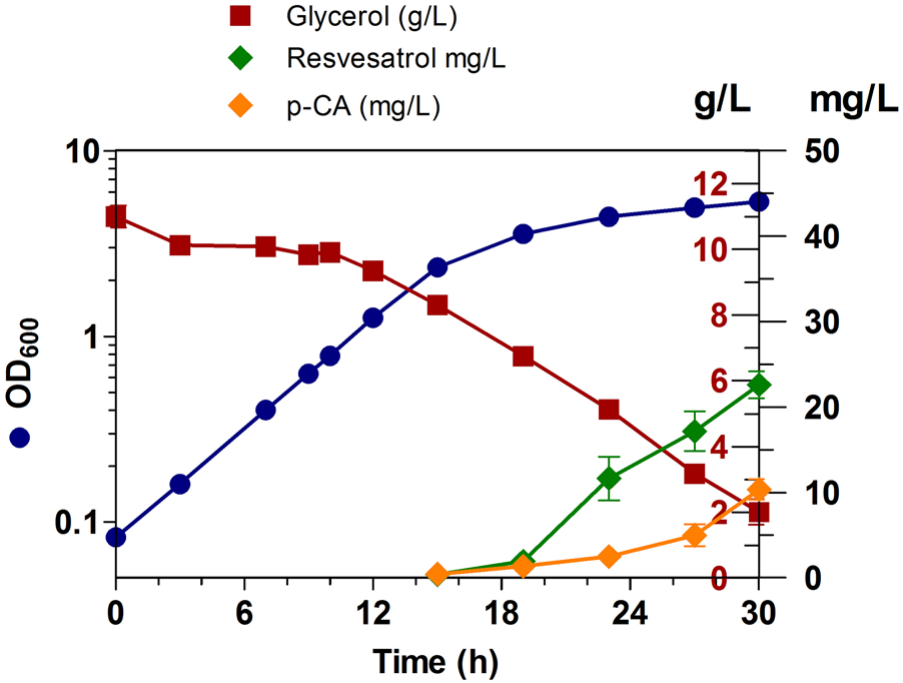
Growth kinetic profile of the coculture of strains W(pheA^−^)Rg and W-Vv. Bioreactor cultures in M9 mineral medium supplemented with glycerol. *Error bars* represent standard deviation

Under coculture conditions, the growth rate was similar to that observed for single strain cultures (Fig. 6; Tables 5 and 6). Both strains were inoculated at a ratio of 1:1 (initial O.D._600_ of 0.1), and the final biomass was 1.78 g/L. Compared to monoculture results, p-coumaric acid accumulation was lower, which can be partially explained considering that this compound is being consumed for resveratrol synthesis. The resveratrol synthesis profile coincided with that of p-coumaric acid production, starting both at 15 h and continuing until the end of the experiment. During the production period that lasted 15 h, glycerol provided carbon and energy for biomass production, p-coumaric acid and resveratrol synthesis. A final resveratrol titer of 22.58 mg/L and 9.16 mg/L of remaining p-coumaric acid were observed at 30 h of total culture time.

**Table 6:** Growth and production parameters for the coculture of strains W(pheA^−^)Rg and W-Vv in glycerol M9 mineral medium, 30 °C

µ (h^−1^)	X_MAX_ (g/L)	Y_X/S_ (g_DCW_/g_S_)	q_S_^a^ (g_S_/g_DCW_ h)	p-CA			
				Y_P/S_ (mg_P_/g_S_)	Y_P/X_ (mg_p_/g_DCW_)	Q_P_ (mg_p_/L h)	p-CA (mg/L)
0.22 ± 0.00	1.96 ± 0.07	0.33 ± 0.10	0.72 ± 0.22	1.16 ± 0.04	5.48 ± 0.18	0.46 ± 0.00	9.16 ± 0.09

				Resveratrol			
				Y_P/S_ (mg_P_/g_S_)	Y_P/X_ (mg_p_/g_DCW_)	Q_P_ (mg_p_/L h)	Resveratrol (mg/L)
				2.86 ± 0.36	18.86 ± 1.28	1.13 ± 0.11	22.58 ± 2.25

^a^Calculated from the exponential growth phase

These results suggest that both the production process and production strains can be improved. By modifying the inoculum ratio of both strains, it would be possible to couple the rate of p-coumaric acid production to its consumption by the resveratrol-producing strain, which has the potential to increase the yield of resveratrol and avoid the toxic effect of p-coumaric acid accumulation [8]. The success of a coculture production system depends on the efficient exchange of molecules between the strains. Transport functions can be modified with the aim of improving the system. The *aaeXAB* operon in *E. coli* is involved in the excretion of various compounds, including p-coumaric acid [22]. It has been shown that overexpression of these genes results in increased tolerance to the toxicity of this aromatic acid [23]. Therefore, it can be expected that overexpression of genes *aaeXAB* in strain W(pheA-)Rg would lead to a higher secretion rate for p-coumaric acid, while the inactivation of this operon in strain W-Vv could result in increased intracellular availability of this precursor for resveratrol synthesis.

As shown in Table 1, diverse strategies have been employed for the generation and cultivation of *E. coli* recombinant strains for resveratrol production. Concerning strain engineering, these approaches have in common the heterologous expression of 4CL and STS enzymes. Further strain improvement modifications involve expressing activities that increase the supply of endogenous metabolic precursors for resveratrol. Cultivation conditions employed for resveratrol production differ concerning media composition. However, with the exception of the present report, p-coumaric acid or l-tyrosine have been supplemented as precursors and a complex medium was used. In the work reporting the highest level of resveratrol (2340 mg/L), in addition to supplementation with p-coumaric acid in a complex medium, the inhibitor cerulenin was employed. In contrast to these reports, it should be noted that the coculture system reported here is based on the use of a minimal medium where glycerol provides all the carbon atoms for resveratrol synthesis. As mentioned above, various strain and culture optimization strategies should enable to further increase resveratrol titer and productivity. In a recent report, a *Saccharomyces cerevisiae* strain was engineered for the production of resveratrol from glucose or ethanol. In fed-batch cultures, a maximum resveratrol titer of 531 mg/L was produced [24]. It remains to be determined if improvement of the *E. coli* coculture system can achieve similar results.

## Conclusions

To the best of our knowledge, this is the first report on the use of a bacterial coculture for the synthesis of resveratrol. This approach opens up the possibility of harnessing the benefits of splitting into two strains a pathway that requires two different precursors belonging to distinct branches of the central metabolism. This strategy can also help to address some toxicity or inhibitory issues due to the intracellular accumulation of intermediaries. Further improvements through metabolic pathway and bioprocess engineering could allow the independent optimization of partial sections of the pathway and the transport activities related to resveratrol synthesis with a potential improvement of the precursor’s supply.

## Methods

### Strains and plasmids

We based this work on the *E. coli* strain W3110, which is a derivative of *E. coli* K-12 [25]. A detailed description of the strains and plasmids used in this work is given in Table 7. Strain W(pheA^−^)Rg is a p-coumaric acid producer, which was previously reported by our group [21]. The genes corresponding to 4CL from *S. coelicolor* A2 (NP_628552.1) and STS from *A. hypogaea* (BAA78617.1) were synthesized by Life Technologies with codon optimization for *E. coli*.

**Table 7:** Strains and plasmids used in this work

Strain	Characteristics	Reference
W3110	*E. coli* F^−^, λ^−^, INV (rrnD^−^ rrnE)1	ATCC 27325
W (pheA^−^)Rg	W3110 (*pheA*::Km) transformed with plasmid pJLBaroG^fbr^ *tktA* and pTrcPALRg	[21]
W-Ah	W3110 transformed with plasmid pTrc-Sc4CL-AhSTS	This work
W-Vv	W3110 transformed with plasmid pTrc-Sc4CL-VvSTS	This work
*Plasmids*		
pMK-RQ-S*c4CL*	Sc4CL synthetized with optimal codon usage for *E. coli*	This work
pMA-*AhSTS*	AhSTS synthetized with optimal codon usage for *E. coli*	This work
pSB3K3-VvSTS	VvSTS synthetized with codon harmonization for E. coli	This work
pTrc99A	Cloning vector carries bla and *lacIq* genes and trc promoter	[32]
pJLBaroG^fbr^tktA	*aroG* ^fbr^ under control of the lacUV5 promoter; and *tktA* under its native promoter, carries lacIq and *tet* genes	[33]
pTrcPALRg	PAL gene from *R. glutinis* gene cloned in pTrc99A	[21]
pTrc-Sc4CL-AhSTS	4CL gene from *S. coelicolor* A2 (Sc) and STS gene from *A. hypogaea (Ah)* cloned in pTrc99A	This work
pTrc-Sc4CL-VvSTS	4CL gene from *S. coelicolor* A2 *(Sc)* and STS gene from *V. vinifera (Vv)* cloned in pTrc99A	This work

The DNA sequence of the STS gene from *V. vinifera* was synthesized by Epoch Life Science Inc. and was codon harmonized for improved protein expression by performing synonymous codon replacement of the original sequence, with the codon having the best frequency match to that in the native organism. This approach was based on a previously published work for codon harmonization [26]. Species-specific codon usage tables were obtained from the Codon Usage Database (http://www.kazusa.or.jp/codon/), and analysis of the codon frequency differences was performed with the website-tool Graphical Codon Usage Analyzer available at http://www.gcua.schoedl.de/index.html [27].

### Plasmids construction

The construction of plasmids pTrc-Sc4CL-AhSTS and pTrc-Sc4CL-VvSTS (Table 7), were designed so that both genes are under the control of the Trc promoter in an operon with a gene encoding 4CL placed next to the promoter and followed by the STS gene. The genes encoding the 4CL from *S. coelicolor* A2 and the STS from *A. hypogaea* were placed each after a ribosome binding site (RBS) taken from the pTrc vector while the STS gene from *V. vinifera* (VvSTS) was designed with a customized RBS sequence using RBS calculator v1.1 [28].

The first construction step consisted on the individual cloning of the 4CL and STS synthesized genes into the vector pTrc99A by digestion with restriction enzymes *Nco*I/*Kpn*I, thus generating the plasmids pTrc-sc4CL and pTrc-ahSTS. The gene AhSTS was then subcloned in the *Kpn*I/*Hind*III sites of pTrc-Sc4CL, producing the plasmid pTrc-Sc4CL-AhSTS. Each plasmid was confirmed by PCR and sequencing. For the construction of the plasmid pTrc-Sc4CL-VvSTS, we started from pTrc-Sc4CL-AhSTS and replaced the sequence of the AhSTS gene with that of the VvSTS gene using restriction sites *Kpn*I/*Hind*III.

### Culture media and conditions

For plasmids construction experiments, solid and liquid Luria–Bertani (LB) media was used, and the antibiotics carbenicillin (Cb; 100 µg/mL), tetracycline (Tc; 30 µg/mL) and kanamycin (Km; 30 µg/mL) were used when required for strain selection.

Cultures for the characterization of production were performed in M9 mineral medium (Na_2_HPO_4_-7H_2_O 12.8 g/L, KH_2_PO4 3 g/L, NaCl 0.5 g/L, NH_4_Cl 1 g/L, MgSO4 249 mg/L, CaCl2 11.1 mg/L, and thiamine 10 μg/L). The carbon source was either glucose or glycerol at 10 g/L for shake-flask experiments and the medium was supplemented with l-phenylalanine 0.3 mM to allow the growth of the *pheA*^−^ mutant strain.

### Culture conditions for strain characterization

Experiments for growth profile characterization were started with an overnight LB culture followed by an adaptation step carried out at a starting density of 0.1 O.D._600_ in a 250 mL baffled shake flasks with 50 mL of M9 medium supplemented with 10 g/L of either glucose or glycerol. Flasks were placed in a shaking incubator at 30 °C and 300 rpm for 12 h. Cells were then harvested and used to inoculate again 250 mL baffled shake flasks with 50 mL of fresh M9 medium at a starting density of 0.1 O.D._600_. Cultures of strains carrying 4CL and STS genes were also supplemented with p-coumaric acid at a concentration of 3 mM. Cultures were incubated at 30 °C, 300 rpm and induced with 0.5 mM of IPTG when they reached 0.8 O.D._600_. Then, cultures continued for another 20 h after induction. All cultures were performed at least in triplicate.

### Coculture fermentations

Batch coculture fermentations were performed in 1 L autoclavable glass bioreactors (Applikon, The Nederlads) connected to an Applikon ADI 1010 BioControlleer and ADI1025 controllers to control pH, temperature, stirrer speed, and aeration rate. The working volume was 750 mL and culture conditions were set at pH 7.0, 30 °C, 600 rpm and an aeration rate of 0.5 vvm of sterile air. pH was controlled automatically by adding a solution of 10 % w/v NH_4_OH.

LB overnight cultures were used to start the inocula in 250 mL baffled shake flasks with 50 mL of mineral M9 medium without addition of p-coumaric acid and supplemented with 0.3 mM l-phenylalanine and 10 g/L glycerol. Cells were then harvested to inoculate bioreactors. Cultures in bioreactors were started in the same fresh medium as the inoculum at an O.D._600_ of 0.05 for each strain, to give a final starting inoculum of 0.1 O.D._600_. Growth was monitored, and when reached 0.8 O.D._600_, cultures were induced at a final concentration of 0.5 mM of IPTG. Cultures continued for 20 h more after induction. Samples of 500 µL of the culture were collected for resveratrol extraction and 1 mL for O.D._600_ and glycerol determination.

### Protein expressions assays

Total protein expression was analyzed using 12 % SDS–polyacrylamide gel electrophoresis and Coomassie blue staining. Briefly, 1 mL aliquots of culture were collected at the end of the cultures and O.D._600_ was determined. All samples were standardized by O.D._600_ (dilution to a volume of 1 mL at 3.0 O.D._600_). Cells were then centrifuged, and the pellet resuspended in 100 µL of distilled water. Then 20 µL were mixed with 10 µL of loading buffer with β-mercaptoethanol and boiled in a water bath at 100 °C for 5 min. Samples of 5 µL each were loaded and resolved on a 12 % SDS–polyacrylamide gel, followed by Coomassie blue staining.

### p-Coumaric acid and trans-resveratrol extractions

To quantify p-coumaric acid and trans-resveratrol, 500 µL aliquots of complete culture were mixed with an equal volume of ethyl acetate. The mixture was vortexed for 30 s and after centrifugation, 400 µL of the organic layer was separated in a clean microfuge tube and evaporated till dryness in an Eppendorf Concentrator. After that, samples were resolubilized in 400 µL of methanol. Finally, samples were filtered through Whatman Anopore inorganic filters of 0.2 µm before injecting into an HPLC system.

### Analytical methods

Cell growth was monitored measuring optical density at 600 nm (O.D._600_) in a spectrophotometer (Beckman DU-70; Fullerton, CA, USA). Glucose and glycerol concentrations were determined using an HPLC system (600E quaternary bomb, 717 automatic injector, 2410 refraction index, Waters, Milford, MA, USA) using an Aminex HPX-87H column (Bio-rad, Hercules, CA, USA). 20 µL of a sample was injected using as mobile phase 5 mM H_2_SO_4_, with a flow rate of 0.5 mL/min. The column temperature was set at 50 °C. Sugars were detected by their refractive index. Identification and quantitation of compounds was performed by comparison of each peak retention time and interpolation in a standard curve. For analysis of p-coumaric acid and trans-resveratrol, we used an HPLC system (Agilent 1100 System, Agilent Technologies, Palo Alto, CA, USA), with a reverse phase column (Phenomenex Synergi Hydro RP C18, 150 × 4.6 mm, 4 um, Phenomenex, Torrance, CA, USA) and equipped with photodiode array detection. We eluted the sample using an isocratic mobile phase of 0.1 % TFA in water (A) and 0.1 % TFA in methanol (B) in a ratio of 60:40 (A:B v/v). Flow was set at one mL/min, column temperature at 45 °C and the injection volume was 10 µL. Identification of p-CA and trans-resveratrol was performed by comparison of the retention times and UV/Vis spectra of each peak with the standards. For quantification of each component, calibration curves were constructed using component standards and by interpolation of the sample signals within the calibration curves.

## Abbreviations

STS: stilbene synthase
4CL: 4-coumaroyl-CoA ligase
PAL: l-phenylalanine ammonia lyase
C4H: cinnamate 4-hydroxylase
TAL: l-tyrosine ammonia lyase
CoA: coenzyme A
DAHP: 3-deoxy-d-*arabino*-heptulosonate 7-phosphate
RBS: ribosome binding site
Cb: carbenicillin
Tc: tetracycline
Km: kanamycin
LB: Luria-Bertani
OD600: optical density at 600 nm
HPLC: high performance liquid chromatography

## References

[1] Renaud S, De Lorgeril M (1992). Wine, alcohol, platelets, and the French paradox for coronary heart disease. Lancet..

[2] Bradamante S, Barenghi L, Villa A (2004). Cardiovascular protective effects of resveratrol. Cardiovasc Drug Rev.

[3] Aggarwal BB, Bhardwaj A, Aggarwal RS, Seeram NP, Shishodia S, Takada Y (2004). Role of resveratrol in prevention and therapy of cancer: preclinical and clinical studies. Anticancer Res.

[4] Joe AK, Liu H, Suzui M, Vural ME, Xiao D, Weinstein IB (2002). Resveratrol induces growth inhibition, S-phase arrest, apoptosis, and changes in biomarker expression in several human cancer cell lines. Clin Cancer Res.

[5] Jeandet P, Delaunois B, Aziz A, Donnez D, Vasserot Y, Cordelier S, et al. Metabolic engineering of yeast and plants for the production of the biologically active hydroxystilbene, resveratrol. J Biomed Biotechnol. 2012. 10.1155/2012/579089 PMC3359829 22654481

[6] Docherty JJ, Fu MM, Tsai M (2001). Resveratrol selectively inhibits *Neisseria gonorrhoeae* and *Neisseria meningitidis*. J Antimicrob Chemother..

[7] Su H-C, Hung L-M, Chen J-K (2006). Resveratrol, a red wine antioxidant, possesses an insulin-like effect in streptozotocin-induced diabetic rats. Am J Physiol Endocrinol Metab..

[8] Watts KT, Lee PC, Schmidt-Dannert C (2006). Biosynthesis of plant-specific stilbene polyketides in metabolically engineered *Escherichia coli*. BMC Biotechnol.

[9] Wu J, Liu P, Fan Y, Bao H, Du G, Zhou J (2013). Multivariate modular metabolic engineering of *Escherichia coli* to produce resveratrol from l-tyrosine. J Biotechnol..

[10] Jeandet P, Vasserot Y, Chastang T, Courot E (2013). Engineering microbial cells for the biosynthesis of natural compounds of pharmaceutical significance. Biomed Res Int.

[11] Leonard E, Yan Y, Fowler ZL, Li Z, Lim CG, Lim KH (2008). Strain improvement of recombinant *Escherichia coli* for efficient production of plant flavonoids. Mol Pharm.

[12] Santos CNS, Koffas M, Stephanopoulos G (2011). Optimization of a heterologous pathway for the production of flavonoids from glucose. Metab Eng Elsevier.

[13] Lim CG, Fowler ZL, Hueller T, Schaffer S, Koffas MAG (2011). High-yield resveratrol production in engineered *Escherichia coli*. Appl Environ Microbiol..

[14] Zecchin A, Stapor PC, Goveia J, Carmeliet P (2015). Metabolic pathway compartmentalization: an underappreciated opportunity?. Curr Opin Biotechnol..

[15] Avalos JL, Fink GR, Stephanopoulos G (2013). Compartmentalization of metabolic pathways in yeast mitochondria improves the production of branched-chain alcohols. Nat Biotechnol..

[16] Zhang H, Pereira B, Li Z, Stephanopoulos G (2015). Engineering *Escherichia coli* coculture systems for the production of biochemical products. Proc Natl Acad Sci.

[17] Zhang H, Li Z, Pereira B, Stephanopoulos G (2015). Engineering *E. coli*-*E. coli* cocultures for production of muconic acid from glycerol. Microb Cell Fact.

[18] Jones JA, Vernacchio VR, Sinkoe AL, Collins SM, Ibrahim MHA, Lachance DM (2016). Experimental and computational optimization of an *Escherichia coli* co-culture for the efficient production of flavonoids. Metab Eng.

[19] Kaneko M, Ohnishi Y, Horinouchi S (2003). Cinnamate : coenzyme A ligase from the filamentous bacterium *Streptomyces coelicolor* A3 (2). J Bacteriol.

[20] Takamura Y, Nomura G (1988). Changes in the intracellular concentration of acetyl-CoA and malonyl-CoA in relation to the carbon and energy metabolism of *Escherichia coli* K12. J Gen Microbiol.

[21] Vargas-Tah A, Martínez LM, Hernández-Chávez G, Rocha M, Martínez A, Bolívar F (2015). Production of cinnamic and p-hydroxycinnamic acid from sugar mixtures with engineered *Escherichia coli*. Microb Cell Fact.

[22] Van Dyk TK, Templeton LJ, Cantera KA, Sharpe PL, Sariaslani FS (2004). Characterization of the *Escherichia coli* AaeAB efflux pump: a metabolic relief valve?. J Bacteriol.

[23] Sariaslani FS (2007). Development of a combined biological and chemical process for production of industrial aromatics from renewable resources. Annu Rev Microbiol.

[24] Li M, Kildegaard KR, Chen Y, Rodriguez A, Borodina I, Nielsen J (2015). De novo production of resveratrol from glucose or ethanol by engineered *Saccharomyces cerevisiae*. Metab Eng.

[25] Bachman BJ (1972). Pedigrees of Some Mutant Strains of *Escherichia coli* K12. Bacteriol Rev.

[26] Angov E, Hillier CJ, Kincaid RL, Lyon JA (2008). Heterologous protein expression is enhanced by harmonizing the codon usage frequencies of the target gene with those of the expression host. PLoS One..

[27] Fuhrmann M, Hausherr A, Ferbitz L, Schödl T, Heitzer M, Hegemann P (2004). Monitoring dynamic expression of nuclear genes in *Chlamydomonas reinhardtii* by using a synthetic luciferase reporter gene. Plant Mol Biol.

[28] Salis HM, Mirsky EA, Voigt CA (2009). Automated design of synthetic ribosome binding sites to control protein expression. Nat Biotechnol..

[29] Beekwilder J, Wolswinkel R, Jonker H, Hall R, De Vos CHR, Bovy A (2006). Production of resveratrol in recombinant microorganisms. Appl Environ Microbioly.

[30] Huang L, Xue Z, Zhu Q. Method for the production of resveratrol in a recombinant bacterial host cell [Internet]. Google Patents; 2007. http://www.google.com.tr/patents/US20070031951.

[31] Katsuyama Y, Funa N, Miyahisa I, Horinouchi S (2007). Synthesis of unnatural flavonoids and stilbenes by exploiting the plant biosynthetic pathway in *Escherichia coli*. Chem Biol.

[32] Amann E, Ochs B, Abel K-J (1988). Tightly regulated tac promoter vectors useful for the expression of unfused and fused proteins in *Escherichia coli*. Gene.

[33] Balderas-Hernández VE, Sabido-Ramos A, Silva P, Cabrera-Valladares N, Hernández-Chávez G, Báez-Viveros JL (2009). Metabolic engineering for improving anthranilate synthesis from glucose in *Escherichia coli*. Microb Cell Fact.

